# Differences between fast and slow muscles in scallops revealed through proteomics and transcriptomics

**DOI:** 10.1186/s12864-018-4770-2

**Published:** 2018-05-22

**Authors:** Xiujun Sun, Zhihong Liu, Biao Wu, Liqing Zhou, Qi Wang, Wei Wu, Aiguo Yang

**Affiliations:** 1 Yellow Sea Fisheries Research Institute, Chinese Academy of Fishery Sciences, Qingdao, 266071 China; 2 Function Laboratory for Marine Fisheries Science and Food Production Processes, Qingdao National Laboratory for Marine Science and Technology, Qingdao, 266200 People’s Republic of China; 3 College of Fisheries, Ocean University of China, Qingdao, 266003 China

**Keywords:** Scallops, Striated adductor muscle, Catch adductor muscle, Proteomics, Transcriptomics

## Abstract

**Background:**

Scallops possess striated and catch adductor muscles, which have different structure and contractile properties. The striated muscle contracts very quickly for swimming, whereas the smooth catch muscle can keep the shells closed for long periods with little expenditure of energy. In this study, we performed proteomic and transcriptomic analyses of differences between the striated (fast) and catch (slow) adductor muscles in Yesso scallop *Patinopecten yessoensis*.

**Results:**

Transcriptomic analysis reveals 1316 upregulated and 8239 downregulated genes in slow compared to fast adductor muscle. For the same comparison, iTRAQ-based proteomics reveals 474 differentially expressed proteins (DEPs), 198 up- and 276 downregulated. These DEPs mainly comprise muscle-specific proteins of the sarcoplasmic reticulum, extracellular matrix, and metabolic pathways. A group of conventional muscle proteins—myosin heavy chain, myosin regulatory light chain, myosin essential light chain, and troponin—are enriched in fast muscle. In contrast, paramyosin, twitchin, and catchin are preferentially expressed in slow muscle. The association analysis of proteomic and transcriptomic data provides the evidences of regulatory events at the transcriptional and posttranscriptional levels in fast and slow muscles. Among 1236 differentially expressed unigenes, 22.7% show a similar regulation of mRNA levels and protein abundances. In contrast, more unigenes (53.2%) exhibit striking differences between gene expression and protein abundances in the two muscles, which indicates the existence of fiber-type specific, posttranscriptional regulatory events in most of myofibrillar proteins, such as myosin heavy chain, titin, troponin, and twitchin.

**Conclusions:**

This first, global view of protein and mRNA expression levels in scallop fast and slow muscles reveal that regulatory mechanisms at the transcriptional and posttranscriptional levels are essential in the maintenance of muscle structure and function. The existence of fiber-type specific, posttranscriptional regulatory mechanisms in myofibrillar proteins will greatly improve our understanding of the molecular basis of muscle contraction and its regulation in non-model invertebrates.

**Electronic supplementary material:**

The online version of this article (10.1186/s12864-018-4770-2) contains supplementary material, which is available to authorized users.

## Background

Adult skeletal muscles are composed of two main classes of fiber types, slow, type I, and fast, type II, which are classified according to myofibrillar ATP staining and immunohistochemistry [1, 2]. Fast and slow muscles, composed of fibers with distinct physiological properties, play indispensable roles in body motion and maintenance of metabolism [3, 4]. Slow and fast muscles occur not only in vertebrates but also in many invertebrates, such as molluscs and crustaceans [5–8]. Over the past decades, extensive work has been done on invertebrate muscles, primarily in general structure of muscles, regulation of muscle contraction, and the mechanisms of contraction and motor function [9–13]. More recently, our knowledge of the unique physical properties of invertebrate muscles, has been reformulated with the advent of structural knowledge of thick and thin filaments [14–19], in vitro motility assay [20, 21], and new evidences on the catch mechanism [22–25]. Recent work on fast and slow skeletal muscles reveals the complex regulatory mechanisms in key components of muscle structure, including the transcriptional and posttranscriptional events [3, 4]. In contrast, invertebrate thick filaments are very different from vertebrate striated thick filaments and show great variation within invertebrates [26, 27]. Muscle-specific genes and proteins in invertebrates (e.g. paramyosin, twitchin, and catchin) have been identified in the past decades, but the regulatory mechanisms for differential expression of these muscle-specific proteins remain largely unknown [26].

Scallops possess fast (striated) and slow (catch) adductor muscles, which lie closely apposed to one another but are divided by a connective tissue sheet. The striated adductor muscle contracts very quickly for swimming, whereas smooth catch adductor muscle lacks striations, contracts for long periods, keeping shells closed with little expenditure of energy [9, 10]. For this reason, scallops are a key model for studies on muscle structure and function.

For scallops, myosin and actin filaments are most abundant in adductor muscles, where they form the sarcomere in fast adductor muscle and the less ordered contractile units in catch muscle [9]. For fast adductor muscle, calcium binds not only to regulatory components of the thin filament but also directly to specific regulatory sites in order to activate the interaction of myosin with actin during phasic contraction (See the review from Chantler [10]). In contrast, twitchin phosphorylation mechanisms in scallop catch muscle may occur in place of direct activation by calcium binding during catch contraction [28]. Although we know the structural and functional distinctions between the two muscles, little information is available on their molecular components and regulatory mechanisms.

**Fig. 1:**
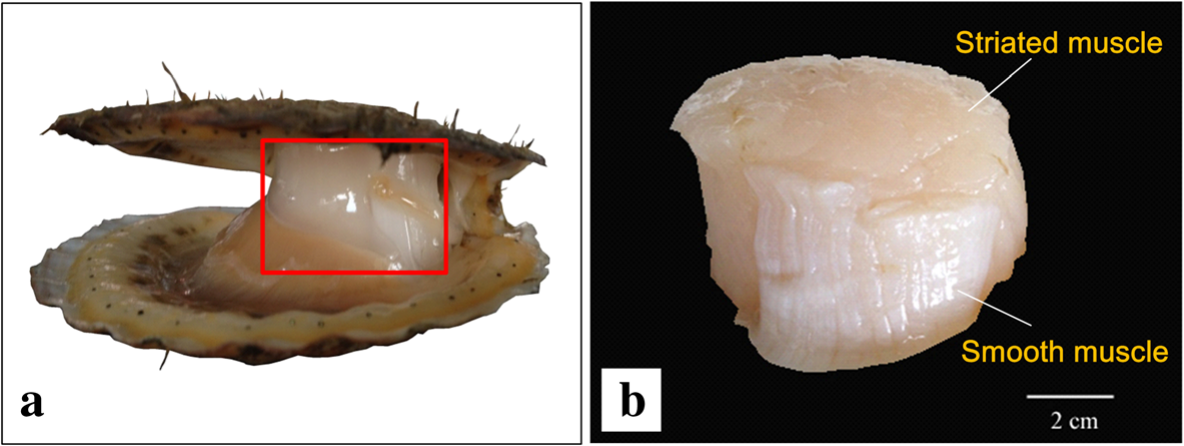
The striated (fast) and catch (slow) adductor muscles of Yesso scallop *Patinopecten yessoensis*. **a**, Photograph of adductor muscles in a live scallop; **b**, Photograph of adductor muscles after removal of all organs and tissues

Our current knowledge on muscle specific proteins in scallops has been established primarily by traditional biochemical and immunohistochemical techniques, which require laborious work and yield a limited number of enzymes or proteins [10]. Recently, the advances in iTRAQ (isobaric tags for relative and absolute quantitation) proteomics and RNA-Seq transcriptomics enable us to identify and quantify thousands of genes and proteins in a single experiment [3, 29–31]. Here, we performed the first, integrated proteomic and transcriptomic analyses of differences between the fast (striated) and slow (catch) adductor muscles in Yesso scallop (*Patinopecten yessoensis*; Fig. 1), with the goal to uncover the molecular components, as well as their regulatory mechanisms in fast and slow adductor muscles of scallops. The comparison of protein and mRNA data in this study provides insights into the regulatory events at transcriptional and posttranscriptional levels that shape the identity of fast and slow adductor muscles. The present results will greatly extend our knowledge on the molecular basis of muscle contraction and its regulation in scallops.

## Results

### Overall statistics for transcriptome and proteome sequencing

Illumina sequencing yielded 93,868,386 raw reads (NCBI accession number: SRR4254476). Filtering low quality reads resulted in clean reads totaling 10.73 Gb, with a GC content of 45.80%. For the four sequencing samples, similar Q20 were observed ranging from 96.83 to 97.16%. After assembly, the transcripts were subsequently assembled into 56,422 unigenes, with the mean length of 584 bp and N50 of 704 bp. The maximum number of unigenes were annotated in Nr (NCBI non-redundant protein) database (18,571), accounting for 32.91% of all unigenes, followed by 25.15% in Swissprot and 21.93% in KOG (euKaryotic Ortholog Groups) database, and 14.74% in KEGG (Kyoto Encyclopedia of Genes and Genomes) database.

Mass spectrometry, iTRAQ proteomics yielded 43,280 unique spectra among the 319,156 spectra detected (ProteomeXchange Consortium dataset identifier PXD005166). Finally, 1591 proteins were identified and annotated in the protein databases. According to Gene Ontology (GO), there were 958 unigenes assigned to three main functional categories, including biological process (BP), cellular component (CC), and molecular function (MF). A total of 3277 unigenes were classified into 26 ortholog groups in KOG database. The pathways determined by KEGG database were grouped into six specific pathways, including metabolism, human diseases, genetic information processing, cellular processes, environmental information processing, and organismal systems.

### Identification of DEGs between the striated and catch muscles

**Fig. 2:**
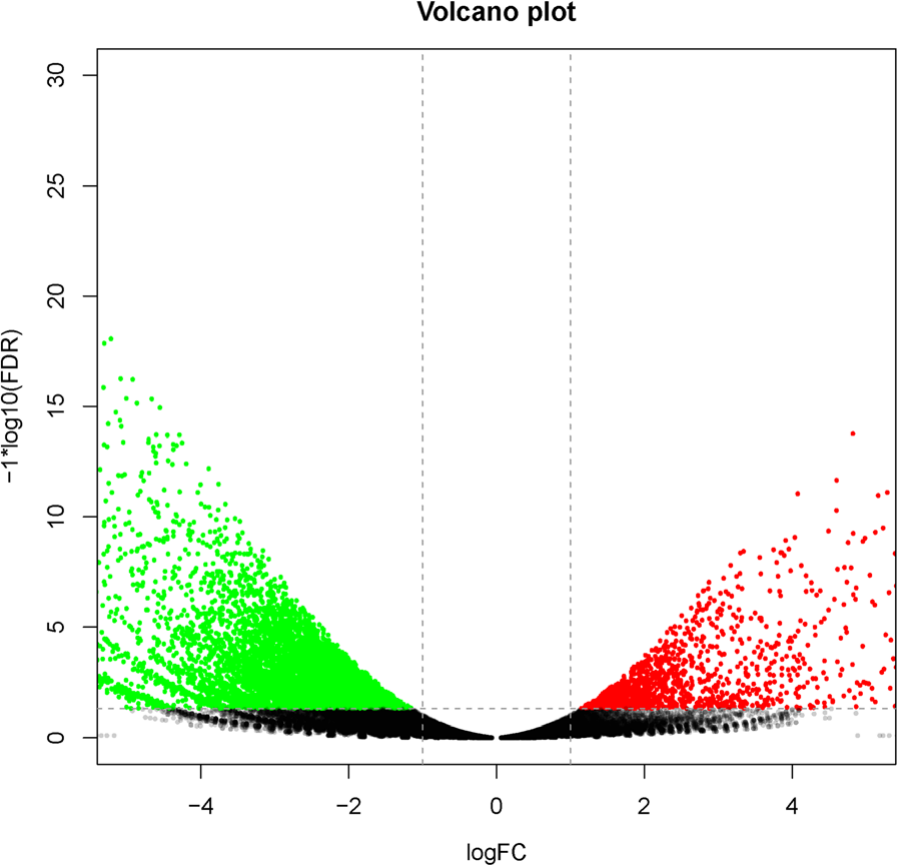
Volcano plots for differentially expressed genes (DEGs) between slow and fast adductor muscles. Points of the plots represent transcripts that are significantly differentially expressed. Green points represent transcripts with significantly lower expression level (slow versus fast muscle), while red circles represent transcripts with significantly higher expression level (*P* < 0.05)

Counts of expressed tags were used to estimate levels of gene expression for replicate samples of catch and striated muscles. Expression levels are tightly correlated between replicates within tissue (Pearson correlation coefficients, 0.9985 and 0.9907, for catch and striated muscle tissues, respectively). Comparison of expression levels between catch and striated muscles produced 9555 differentially expressed genes (DEGs), with the filter criteria of false discovery rate (FDR) < 0.05 and |log2FoldChange| > 1. Among the DEGs, there were 1316 upregulated and 8239 downregulated genes identified between catch and striated muscles. Volcano plot illustrates the asymmetry in differentially expressed genes (Fig. 2) between downregulated (green) and upregulated (red) genes.

**Fig. 3:**
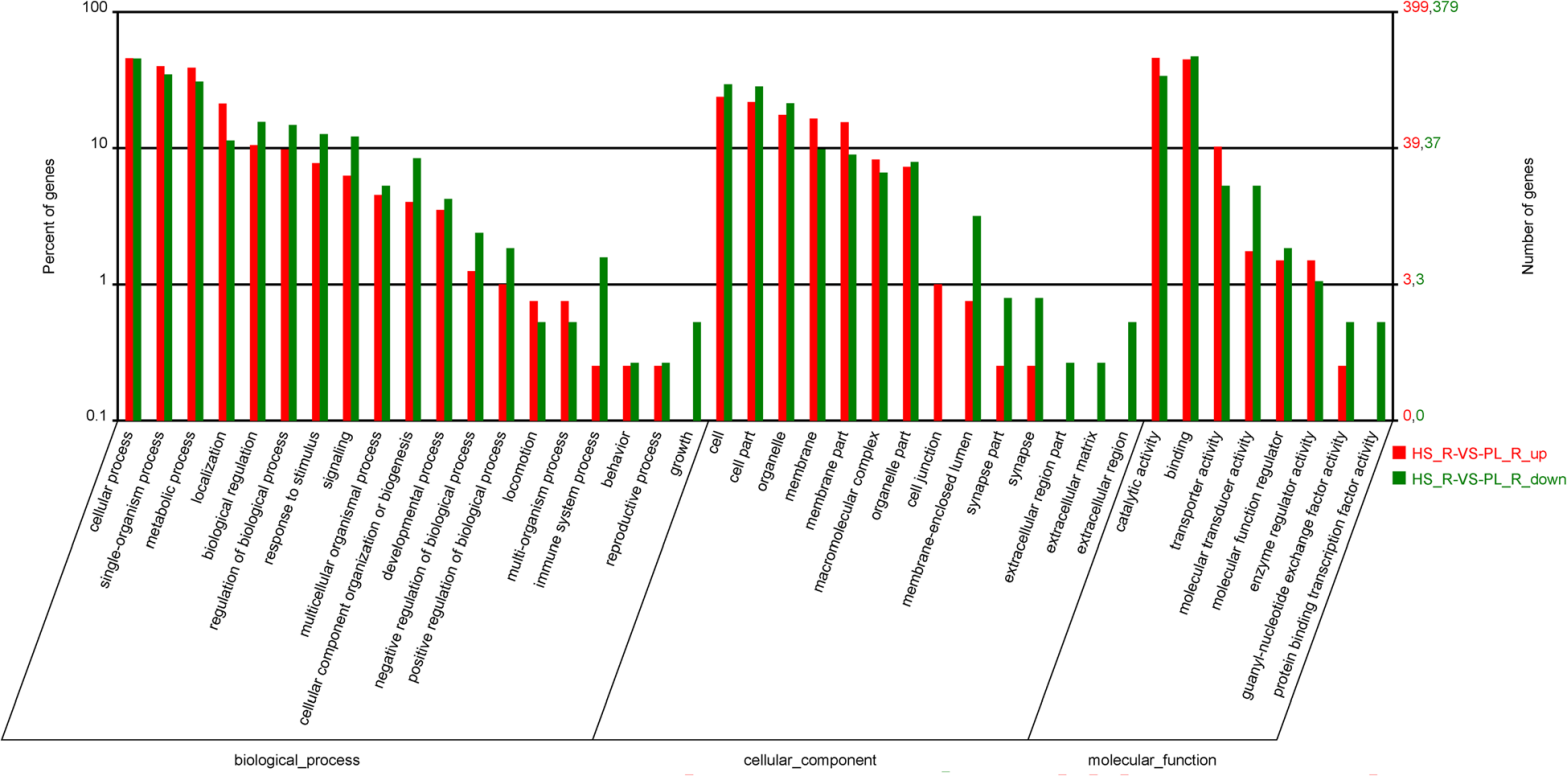
Gene Ontology (GO) enrichment results for DEGs between slow and fast muscle. HS, catch (slow) muscle; PL, striated (fast) muscle. Red bars represent upregulated genes for slow versus fast muscles, while green bars represent downregulated genes in the same comparison

DEGs were assigned to 41 sub-categories of GO terms (Fig. 3). Most of the unigenes were assigned to cellular process, localization, metabolic process, single-organism process, cell, cell part, binding, and catalytic activity. The DEGs were further subjected to KEGG pathway analysis, which were classified into 222 signaling pathways. The most enriched pathways include calcium signaling pathway, protein digestion and absorption, vascular smooth muscle contraction, ECM (extracellular matrix)-receptor interaction, cardiac muscle contraction, glycolysis/gluconeogenesis, regulation of actin cytoskeleton, MAPK signaling pathway and hedgehog signaling pathway.

**Fig. 4:**
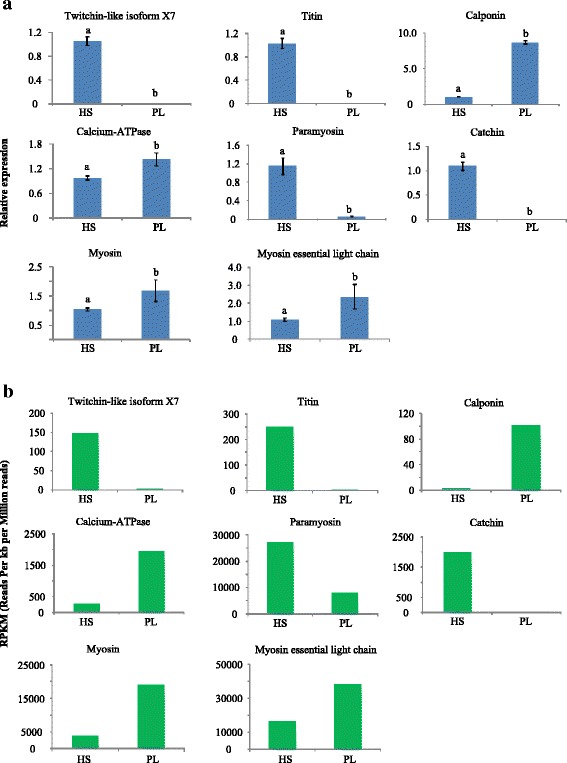
Quantitative RT-PCR (qPCR) results of muscle-related genes between slow and fast muscles of *P. yessoensis*. **a**, the relative expression obtained from qPCR analysis; **b**, the RPKM (reads per kb per million reads) values of the selected genes according to transcriptomic data. HS, catch (slow) muscle; PL, striated (fast) muscle

Eight DEGs related to muscle structure and function, including twitchin-like isoform X7, titin, calponin, calcium-ATPase, paramyosin, catchin, myosin, and myosin essential light chain, were selected to verify the RNA-Seq results by the quantitative RT-PCR (qPCR). Compared with the striated muscle, the catch muscle had a significantly higher expression of twitchin-like isoform X7, titin, paramyosin, and catchin, whereas the other four genes had a significantly lower expression (*p* < 0.01; Fig. 4a). According to the transcriptomic data, the RPKM (Reads per kb per million reads) values of the selected genes were summarized in Fig. 4b, which showed that the expression pattern of these genes was all in accordance with the qPCR results.

### Identification of DEPs between the striated and catch muscles

The iTRAQ-based quantitative proteomics identified 474 differentially expressed proteins (DEPs) between the slow and fast muscles, including 198 upregulated and 276 downregulated DEPs (Additional file 1: Table S1). These DEPs include dozens of muscle-specific proteins, such as calmodulin, myosin essential light chain, myosin heavy chain (MHC) II, titin, tropomodulin, troponin C, myosin essential light chain, paramyosin, gelsolin-like protein, myopalladin, galponin, myophilin, and twitchin. Furthermore, quantitative proteomics revealed many proteins related to calcium signaling, sarcomere and cytoskeleton, including sarcoplasmic calcium-binding protein, smoothelin-like protein, actinin, filamin, actin-interacting protein, PDZ and LIM domain protein.

Similarly, the most enriched KEGG pathways include ECM-receptor interaction, glycolysis/gluconeogenesis, cardiac muscle contraction, protein digestion and absorption, regulation of actin cytoskeleton, calcium signaling pathway, vascular smooth muscle contraction, hedgehog signaling pathway, etc. In the pathway of ECM-receptor interaction, ECM related proteins, such as collagen, laminin, and perlecan, were expressed significantly higher in catch muscle as compared with striated muscle.

### Association analysis of transcriptome and proteome

**Fig. 5:**
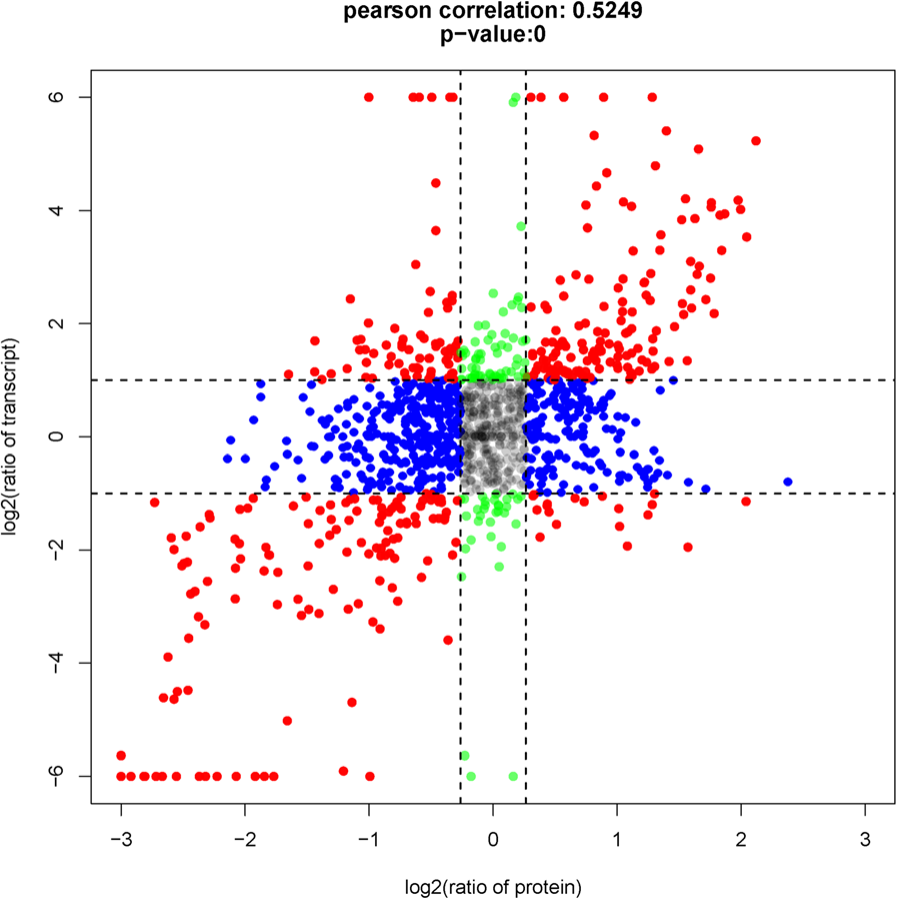
Association analysis of transcriptome and proteome differences between slow and fast muscles in *P. yessoensis* (protein fold changes > 1.2 and mRNA fold change > 2). Red dots (group 1, 3, 7, and 9) represent significant changes of expression in both mRNA and protein; Green dots (group 2 and 8) show significant changes in mRNA expression levels only; Blue dots (group 4 and 6) denote significant changes of expression in protein levels only; Grey dots (group 5) display no significant change of expression in either mRNA or proteins

Association analysis of transcriptome and proteome data for slow versus fast adductor muscles revealed a nonlinear relationship between mRNA and protein expression, with the Pearson’s correlation coefficient of 0.5249 (Fig. 5). The colored dots representing 1236 differentially expressed unigenes (slow vs. fast muscle) were unevenly distributed in nine groups. Red dots in groups 3 and 7 represent the elevated expression of mRNA and protein levels in fast (group 3) and slow (group 7) muscles; Red dots in groups 1 and 9 show the opposite changes of mRNA levels and protein abundances; Green dots (group 2 and 8) indicate changes of mRNA expression levels only; Blue dots (group 4 and 6) denote changes of expression in protein levels only; Grey dots (group 5) display no significant change of expression in either mRNA or proteins.

**Fig. 6:**
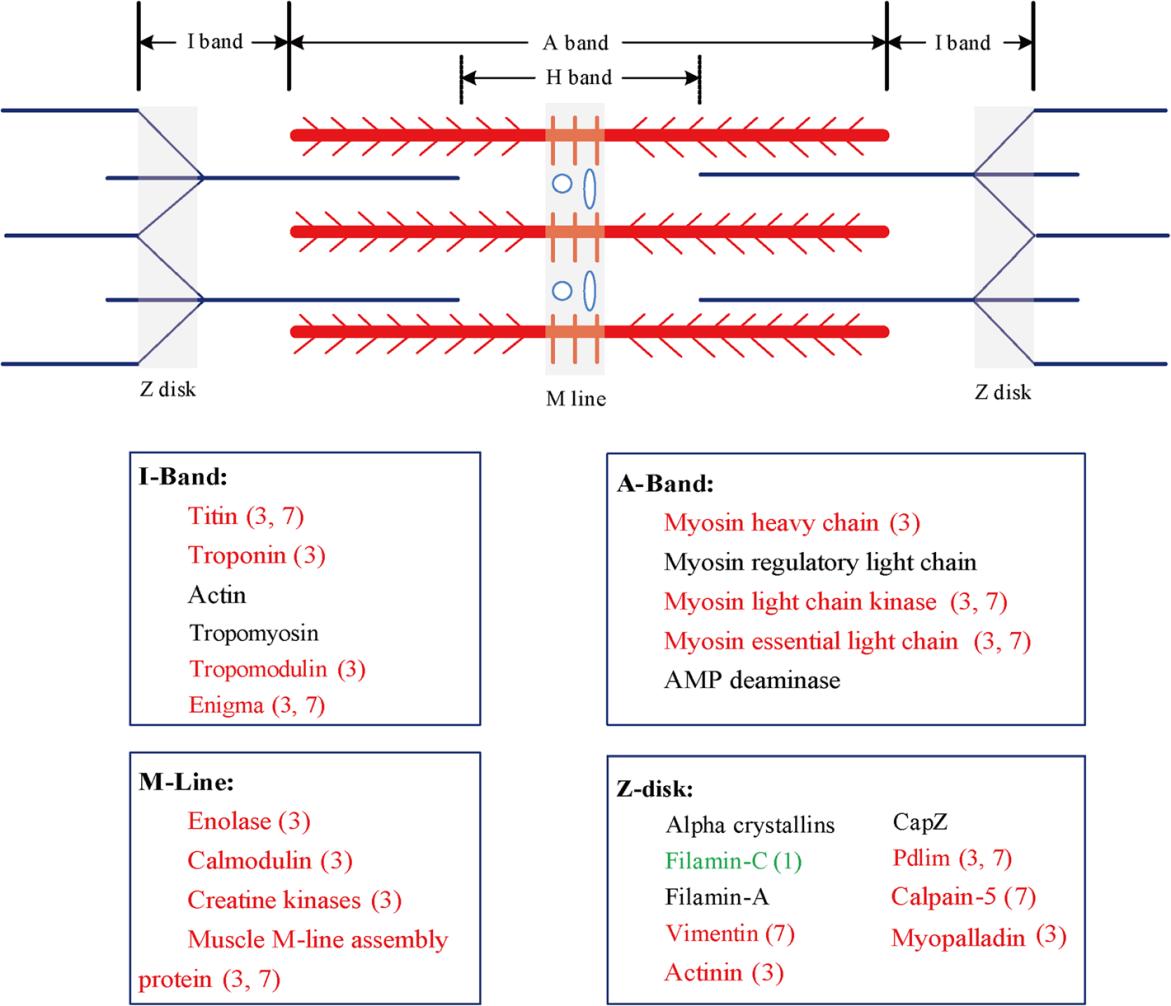
Schematic representation of fiber structure and composition for striated (fast) adductor muscle in *P. yessoensis*, based on its vertebrate counterparts. Localization of proteins identified was predicted from transcriptomic and proteomic analyses. Red fonts represent those proteins with significantly higher expression in fast muscle; Green fonts represent proteins with significantly lower expression in fast muscle; Black fonts represent no quantitative difference in protein levels between slow and fast muscles. Numbers in parentheses are the group distributions, according to the association analysis of transcriptome and proteome data

In groups 3 and 7, the similar changes in mRNA and protein levels suggest that expression of these proteins in the two muscles is regulated at the transcriptional level. 281 unigenes (22.7%) showed a positive relationship between protein abundances and mRNA enrichment in the striated and catch muscles, including myofibrillar proteins, sarcoplasmic reticulum (SR) proteins, metabolism related enzymes, and membrane and extracellular proteins (Additional file 2: Table S2). In group 3, the identified unigenes mainly represent proteins enriched in striated muscle, such as myosin, actinin, calmodulin, enolase, titin, tropomodulin, and troponin. These muscle proteins were predicted to locate in the sarcomere, as seen in vertebrate striated muscles (Fig. 6). Significantly higher expression of the main components of myosin, including myosin heavy chain (MHC) and myosin essential light chain (E-LC), was detected in striated muscle compared to catch muscle. The conventional (class II) and unconventional myosin classes were found to be expressed in a tissue-specific manner. The MHC class II consistently expressed at a much higher level in striated muscle than in catch muscle, whereas unconventional myosin classes (e.g. unconventional myosin-XVI-like isoform X3 and non-muscle myosin) showed significantly higher expression in catch muscle than in striated muscle. Three troponin subunits, troponin C, troponin I and troponin T, also showed higher expression, at mRNA and protein levels, in striated muscle compared to catch muscle. Moreover, three important sarcoplasmic reticulum (SR) proteins, sarcoplasmic/endoplasmic reticulum calcium ATPase (SERCA), calcium-transporting ATPase, and sarcoplasmic calcium-binding protein (SCP), showed significantly higher mRNA and protein expression in striated muscle than in catch muscle. In addition, many proteins involved in calcium signaling pathway and metabolic pathways were also detected in group 3, such as calcium binding protein and arginine kinase. Moreover, unigenes encoding key enzymes involved in glycolysis were detected in group 3, such as glycogen phosphorylase, glycogen debranching enzyme and pyruvate kinase. The levels of all of these enzymes were positively correlated with mRNA levels in striated muscle. For arginine kinase, mRNA and protein expression showed approximately 4.5-fold and 3-fold higher expression in striated muscle than in catch muscle. Identified unigenes in group 7, in contrast to those in group 3, mainly represent proteins enriched in catch muscle, including paramyosin, twitchin, catchin, calponin, gelsolin-like protein, myophilin, smoothelin-like protein, and titin. Overall, expression patterns of mRNA and proteins were similar in most of the myofibrillar proteins, except for actin, tropomyosin, AMP deaminase, filamin, crystallins, and CapZ (Fig. 6).

In contrast, 658 unigenes (53.2%), in groups 1, 2, 4, 6, 8 and 9, display the striking differences between protein and mRNA expression levels, which suggest the existence of regulatory events at the posttranscriptional level. For instance, 89 unigenes in groups 1 and 9 had a negative correlation of mRNA expression and protein abundances, such as filamin, 26S proteasome, ATP-dependent RNA helicase, calpain, E3 ubiquitin-protein ligase, collagen, laminin, etc. Additionally, 473 genes (blue dots) in groups 4 and 6 showed elevated protein levels in either the catch or striated muscles, but with no difference in mRNA expression. They were mainly associated with collagen, ribosomal proteins, glycogen synthase and mitochondrial genes. Finally, 96 unigenes in groups 2 and 8 (green dots), such as insulin-like growth factor, glycogen synthase, AMP deaminase, 26S protease regulatory subunit, and lamin, showed elevated mRNA levels in either the catch or striated muscles but had similar protein expression in the two muscles.

## Discussion

In this study, we performed quantitative transcriptomic and iTRAQ-based proteomic analyses of differences between the striated and catch adductor muscles of Yesso scallop *P. yessoensis*. Proteomic analysis quantified more than 1500 proteins, comprising the largest quantitative data set to date for proteins in scallop adductor muscles. The discovery of ~ 500 DEPs and 9555 DEGs greatly extends our knowledge on the molecular components and complex regulatory events in molluscan muscle physiology. We summarize our findings below about some of the major molecular components of the striated and catch adductor muscles, and highlight that regulatory mechanisms at the transcriptional and posttranscriptional levels are essential in the maintenance of muscle structure and function in scallops.

### Myosin heavy chain

Scallop adductor myosin is a regulatory myosin, which possesses the necessary machinery within its own structure to control its interaction with actin by the cross-bridge cycle [10]. Similar to other conventional myosins, the scallop myosin is composed of two heads with myosin heavy chains (MHCs), followed by a neck domain to which the light chains (LCs) bind, and a long coiled-coil tail. A recent study revealed that the myosin heads in Ca^2+^-regulated myosin filaments of scallop striated muscles interact in a similar way to those in phosphorylation-regulated filaments of vertebrate smooth muscles [17]. In this study, relatively higher expression levels of E-LC and R-LC are detected in the striated muscle as compared with the catch muscle, which may correspond to high actin-dependent MgATPase activity in the striated muscle.

Scallop muscles have a similar myosin-linked regulation mechanism as vertebrates, because they share a common structural basis for switching off thick-filament activity in relaxed muscles [10, 17]. However, unlike their vertebrate counterparts, the striated and catch muscle MHCs in scallop myosin heads are expressed from a single gene as alternatively spliced products [32]. Both conventional (Class II) and unconventional myosin classes were detected in the mantle tissue of scallop *P. yessoensis* in previous studies [33–35], as well as in the present study. However, the mantle MHC II is similar to vertebrate non-muscle MHC II rather than scallop muscle MHC II, having only 22% sequence identity with scallop muscle MHC II at the amino acid level. Similarly, we also found the extremely low sequence identity (< 20%) between the unconventional MHC and conventional MHC sequences. In the present study, four MHC mRNA variants produced from a single gene, in groups 2, 3 and 6, display the differential expression between mRNA levels and protein abundances, which indicate that regulation events may occur at the posttranscriptional level, such as alternative RNA splicing.

### Troponin

Troponin is the tropomyosin-binding protein, a central element in the thin-filament-linked Ca^2+^ regulatory system of vertebrate striated muscles [36–38]. It is often composed of three subunits: troponin C, troponin I and troponin T. As in vertebrate striated muscle, we detect an equal ratio of C:I:T troponin subunits in both striated and catch muscles. The high expression of three troponin subunits in the striated muscle supports the prevailing view being that scallop striated muscles possess some form of thin filament-linked regulation [10]. However, we find that sequences of scallop troponin subunits exhibit low (< 30%) homology with their vertebrate counterparts, as reported in previous studies [39, 40]. Thus, the different structures and functional adaptations of the three molluscan troponin subunits suggest a very different mechanism of action to that seen in vertebrates. As indicated in molluscs, troponin may regulate contraction through activating mechanisms involving the structural troponin C binding site as well as the inhibitory region of troponin I [10, 41]. In the present study, troponin C and T show the increased mRNA and protein expression in striated muscle compared to catch muscle, while troponin C exhibit significant changes of expression in protein levels only. The distinct protein and isoform expression of troponin in striated and catch muscles suggests the existence of fiber-type specific, posttranscriptional regulatory mechanisms.

### Titin

Titin is an exceptionally large protein in vertebrate muscles, which can link filamin and α-actinin together in the Z-line periphery of the striated muscle, or in dense bodies of the smooth muscle [42, 43]. In the present study, multiple isoforms of titin are enriched in either the striated or catch muscle (seven dots in group 3 and three dots in group 7; Fig. 5), which are probably generated by alternative RNA splicing from the same gene, as suggested in vertebrate muscles [42, 44]. Therefore, the differential protein and isoform expression of titin in striated and catch muscles may be regulated by complex mechanisms, including the transcriptional and fiber-type specific, posttranscriptional regulatory events.

### Other striated and catch muscle components

The similar regulations of mRNA and protein abundances are found in many muscle-specific components, which indicate that the regulatory events occur at the transcriptional level. Calponin is responsible for cross-linking actin and myosin filaments and can organize the contractile filaments into a three-dimensional network for the proper orientation and spatial distribution during force development in vertebrate smooth muscles [45]. In contrast to vertebrate, calponin in molluscan catch muscle is thought to be involved in catch regulation, serving as a competitive inhibitor of actomyosin ATPase [24, 46]. It is evidenced that the abundance of calponin in catch muscle is predominately regulated by the transcription activation of mRNA according to the present results. Moreover, smoothelin-like 1 protein (SMTNL1) may act as a physiological regulator of muscle contraction through cAMP-activated kinases in vertebrate smooth muscles [47]. In this study, SMTNL1 is enriched in catch muscle by the transcriptional regulation and may play some indirect roles in catch muscle contraction. In addition, actinin is localized at dense bodies in the smooth muscle and Z-disk in striated muscle of vertebrates, where it forms a lattice-like structure and stabilizes the muscle contractile apparatus [48–50]. It is suggested that the enrichment of actinin protein in striated muscle of *P. yessoensis* by transcriptional regulatory events may be devoted to cross-link actin filaments from adjacent sarcomeres during Z-disk assembly. Another example for the transcriptional regulation in muscle components is sarcoplasmic reticulum (SR) proteins. As indicated in vertebrates, SR proteins in striated muscle is specialized for releasing Ca^2+^, following sarcolemma depolarization, in order to activate muscle contraction [51]. For scallops, the striated adductor muscle has twice as much of its surface covered with sarcoplasmic reticulum as catch muscle, which is likely associated with a higher contraction rate and reduced relaxation time in striated cells [52]. The present results reveal that the elevated mRNA and protein levels of SR-related genes (e.g. SERCA, calcium-transporting ATPase, and SCP) in striated muscle are mainly controlled by the transcriptional regulatory events. The abundance of SR proteins in striated adductor muscle of scallops may be responsible for the increasing uptake of calcium and maintenance of calcium homeostasis during fast contraction and relaxation cycles [52].

Besides the transcriptional regulatory events, the striking differences between protein abundances and mRNA expression provide evidences for regulation events at the posttranscriptional level in striated and catch adductor muscles. For instance, filamin, an actin-binding protein, may be involved in reorganizing the cytoskeleton in response to signaling events and fulfill structural functions at the Z-disk in vertebrate muscles [53, 54]. In the present study, protein abundances of filamin in catch muscle (in groups 1 and 4; Fig. 5) may be involved in the slow contraction and relaxation peculiar to catch muscle, which are probably achieved by the regulatory events at the posttranscriptional level.

### Implications for catch mechanism

Catch is a mechanism found in molluscan catch muscles, in which tension is maintained at a relatively low energy cost [10, 55]. In this study, a variety of proteins preferentially expressed in the catch muscle, such as paramyosin, twitchin, catchin, and other muscle specific proteins, shed potential lights on the molecular components of catch regulation in scallops. As summarized by a recent review [10], three main theories have been put forward as possible explanations for the catch mechanism. First, the unique structure of paramyosin-rich thick filaments is thought to be responsible for direct interactions between adjacent thick filaments [26, 56, 57]. Although some new evidence shows that paramyosin may not play a direct role in the maintenance of catch, an indirect role, such as the formation of a rigid network of inter-myofilament connections [22], is more difficult to exclude. A second theory holds that catch involves the formation of a long-lived actomyosin state [58].

More recently, new theories have arisen to take into account the unique roles for catchin and twitchin in the catch mechanism [59–61]. Catchin has been determined as an alternatively spliced product of the MHC gene with a unique non-helical N-terminal sequence, which is identical with the C-terminal 830 residues of the MHC [62]. It has been hypothesized that its unique globular N-terminus could be involved in tethering actin while the MHC-derived C-terminal coiled-coil interacts with myosin or paramyosin at the core [10]. Similarly, we reveal that the enrichment of catchin mRNA and protein levels in catch muscle compared to striated muscle in *P. yessoensis* indicates a potential role of catchin in catch regulation at the transcriptional level.

Twitchins are giant kinase molecules found in both the striated and catch muscles, where they are located within the A-band and at the A-I junction. In the unphosphorylated state, twitchins could interact directly with myosin, paramyosin, and catchin during the catch state [60, 63]. A previous study shows that twitchin mRNA is expressed at higher levels in striated muscle than in catch muscle [64]. However, we show that expression of twitchin proteins is significantly higher in catch muscle than in striated muscle, which supports the central role of twitchin in the catch mechanism. More interestingly, we find that three mRNA variants encoding twitchin, in groups 1, 4 and 7 (Fig. 5), are resulted from alternative splicing of the same gene in catch muscle. The striking differences of twitchin between protein and mRNA expression levels reflect the existence of regulatory events at the transcriptional and posttranscriptional levels. The present study reveals, for the first time, the fiber-type specific, posttranscriptional regulation may be involved in the maintenance of catch muscle structure and catch regulation.

### Implications for energy metabolism of striated and catch muscles

The regulatory events at the transcriptional and posttranscriptional levels are not only found in the muscle-specific genes and proteins, but also in key enzymes involved in glycogen synthesis and glycolysis. A previous study indicates that most ATP equivalents are derived from arginine phosphate, in the striated muscle during the snap response, while the energy is mainly supplied from glycolysis, in the catch muscle during valve closure [65]. Arginine phosphate is the major fuel powering phasic contractions by the striated muscles of scallops, which can generate approximately 70% of the ATP used for phasic contractions [66–68]. As indicated, arginine kinase catalyses the reversible conversion of ADP and arginine phosphate into arginine and ATP, the activities of which reflect reliance on rapid initial bursts of phasic contraction [65, 66]. In this study, the central role of arginine phosphate in supporting phasic contraction is therefore supported by the transcriptional regulation of arginine kinase in striated muscle. Furthermore, the similar regulation of mRNA expression and protein abundances for glycolytic enzymes suggests that catabolic processes in relation to valve snap and closure responses are predominantly regulated by the transcriptional activation of the metabolic genes. However, the striking differences of glycogen synthase between protein abundances and mRNA expression levels (in groups 2 and 6; Fig. 5) indicate the existence of the posttranscriptional regulatory event in glycogen synthesis of striated and catch muscles. Overall, it is therefore suggested that energy metabolism in scallop adductor muscles is controlled by complex mechanisms, including the transcriptional and posttranscriptional regulatory events.

## Conclusions

The integrated proteomics and transcriptomics study reveals a number of muscle-specific genes and proteins in striated and catch adductor muscles of Yesso scallop *P. yessoensis*, including muscle contractible proteins, membrane and extracellular matrix, and enzymes of metabolic pathways. The comparison of protein and mRNA data in this study provides the insights into complex regulatory events at the transcriptional and posttranscriptional levels that shape the identity of fast and slow adductor muscles. The striking differences between protein abundances and mRNA expression levels support the existence of fiber-type specific, posttranscriptional regulatory mechanisms in striated and catch adductor muscles. These findings will greatly improve our understanding of the molecular basis of muscle contraction and its regulation in non-model invertebrates.

## Methods

### Biological material

Two-year-old live individuals of *P. yessoensis* (*n* = 6) were obtained from a commercial hatchery in Yantai, China. To obtain high quality of gene expression data, all of these scallops were held under the same conditions in seawater at 16 ± 2 °C for three days at YSFRI (Yellow Sea Fisheries Research Institute). Scallops were fed with *Isochrysis galbana* and two-thirds of the culture water was exchanged every day. The striated and catch adductor muscles were separately dissected from each scallop and frozen and stored in liquid nitrogen individually. Two biological replicates for transcriptomic and proteomic analyses were prepared by dividing the same muscle sample into two parts for total RNA and proteins extraction, respectively.

### RNA-Seq library construction, sequencing and quality control

Total RNA was isolated from the collected adductor muscles with Trizol Reagent (Invitrogen), following manufacturer’s instruction. RNA purity and quality were checked using a NanoPhotometer™ spectrophotometer (Implen, CA, USA) and 1% agarose electrophoresis. Extracted mRNA was enriched by Oligo(dT) beads, with removal of rRNA using a Ribo-ZeroTM Magnetic Kit (Epicentre). Then the enriched mRNA was fragmented into short fragments and reverse transcribed into cDNA with random primers. After synthesis of second-strand cDNA, fragments were then purified, end repaired, polyadenylated, and ligated to Illumina sequencing adapters. Ligation products were size-selected by agarose gel electrophoresis and sequenced by Gene Denovo Biotechnology Co. (Guangzhou, China), using Illumina HiSeqTM 2500. Low quality reads were removed, according to the previous study [31].

### De novo assembly and annotations

De novo assembly the transcriptome was carried out with the short-read assembling program Trinity [69]. The unigene expression was calculated and normalized to RPKM (Reads Per kb per Million reads). To annotate the unigenes, we used the BLASTx program [70] with an E-value threshold of e^− 5^ to NCBI non-redundant protein (Nr) database (http://www.ncbi.nlm.nih.gov) [71], the Swiss-Prot protein database (http://www.expasy.ch/sprot) [72], the Kyoto Encyclopedia of Genes and Genomes (KEGG) database (http://www.genome.jp/kegg) [73], and the COG/KOG database (http://www.ncbi.nlm.nih.gov/COG) [74]. GO annotation of unigenes was analyzed by Blast2GO software.

### Analysis of differentially expressed genes (DEGs) and qPCR validation

Clean reads of expressed data were mapped to the reference transcriptome using the short-read alignment tool, Bowtie2, with default parameters [75]. We used the edgeR package [76] to identify differentially expressed genes between the two muscle types, as those with a significant fold-change of ≥2 in expression level and a false discovery rate (FDR) < 0.05. All DEGs were mapped to GO terms in the Gene Ontology database (http://www.geneontology.org/) [77]. The enriched GO terms were selected with FDR (False discovery rate) < 0.05. KEGG pathway enrichment analysis was also performed to identify significantly enriched metabolic pathways or signal transduction pathways in DEGs at the same condition.

To verify data accuracy of high-throughput sequencing, quantitative real-time PCR (qPCR) was used to compare the relative mRNA expression of significantly expressed genes between the striated and catch adductor muscles. Total RNAs were extracted from the striated and catch muscles using Trizol Reagent (TAKARA). cDNA was synthesized, using the Bestar qPCR RT Kit, and Real time PCR was performed using DBI SybrGreen qPCRmasterMix according manufacture’s instructions on a Mx3000P (Agilent Stratagene). In this study, β-actin was selected as the reference gene because its RPKM values were stable among samples according to the transcriptomic data. The qPCR analysis was performed with three biological and three technical replicates. The comparative Ct method (2^-△△Ct^ method) was used to calculate the relative gene expression of the samples, which was normalized to β-actin mRNA level. The expression data were subsequently subjected to independent t-test in SPSS 17.0 to determine whether there was any difference significant at the *P* < 0.05 level.

### Protein extraction, digestion, and iTRAQ analysis

Total protein was extracted from adductor muscles by the cold acetone method, following previous studies [78, 79]. Briefly, muscle samples were subjected to a mixture of EDTA (Ethylene Diamine Tetraacetic Acid), 1 mM PMSF (Phenylmethanesulfonyl fluoride), and 10 mM DTT (DL-Dithiothreitol) and were the ground to disrupt the cells. After centrifugation at 25,000×g for 20 min at 4 °C, 10 mM DTT was added and incubated at − 20 °C for overnight. The remnant pellet was treated with 10 mM DDT in 1.5 ml cold acetone, followed by centrifugation and air drying. The obtained pellet was then suspended and centrifuged for 20 min at 25,000×g. Next, 10 mM DDT and 55 mM IAM was added to the sample, which was incubated in a dark room for 45 min, followed by another step of suspension. The protein was measured using the 2-D Quant Kit (General Electric Company, USA) and confirmed by SDS-PAGE method according to Qin et al. [78].

Proteins were labeled, using the iTRAQ labeling kit (Applied Biosystems) according to manufacture’s instructions with minor modification. Digested protein products were then dried, dissolved and labeled. The labeled striated and catch adductor samples were fractionated using an SCX column on an HPLC system (LC-20AB, Shimadzu, Japan). The fractionation procedure for each sample was performed as in the previous study [79]. Briefly, peptides were dissolved and then flowed into columns (1 mL/min), followed by elution using buffer A (25 mM NaH_2_PO4 and 25% ACN) and Buffer B (a mixture of Buffer A and 1 M KCl).

The peptides were injected and separated in a Nano-LC system, using a C18 analytical reverse-phase column (300 nL/min). The column was equilibrated with a gradient schedule of different concentrations (5, 45, 80, and 5%) of Solution B (a mixture of 95% acetonitrile and 0.1% formic acid [78]). A Triple TOF 5600 instrument was used for the mass spectrometry. All spectrometry data were collected and summarized using Bruker Daltonics micrOTOFcontrol. The raw data from the LC-MS/MS were transformed into MGF files and analyzed using the Data Analysis Software. The Mascot search engine was set to the following conditions, such as trypsin (digestion enzyme), cysteine carbamidomethylation (fixed modification), glutamine (pyroglutamic acid), and iTRAQ 8Plex on tyrosine (variable modification). The scanning range for MS was from 50 to 2000 m/z, with nitrogen as the collision gas (voltage, 1250 V; interface temperature, 150 °C). These experimental procedures were performed by Guangzhou Gene denovo Biotechnology Co., Ltd. (Guangzhou, China).

### Functional analysis of differentially expressed proteins (DEPs) and its association analysis with transcriptome

To annotate the differentially expressed proteins, WEGO and Blast2GO were both used to search against the GO database according to the previous studies [78, 79]. KEGG pathway analysis was also performed to identify significantly enriched metabolic pathways in the striated or catch adductor muscles of scallops. A significance threshold of 0.05 was selected for the functional annotation of DEPs in the two muscles.

We used Pearson correlation to assess the relationships between mRNA and protein expression levels in the striated and catch muscles. The log2 transformation of the average fold changes was obtained for the transcriptome and proteome data. The transformed data was calculated and displayed as the scatter plots using R program, with the screening criterions (for mRNA, fold change > 2; for proteins, fold change > 1.2).

## Additional files

Additional file 1: **Table S1.** The identification and annotation results of differentially expressed proteins (DEPs) between the striated and catch adductor muscles by the iTRAQ-based quantitative proteomics. There are a total of 474 DEPs identified between the two muscles, which include 198 upregulated and 276 downregulated DEPs. The functional annotation for these DEPs is associated with significantly enriched GO terms and KEGG pathways. (XLSX 135 kb)

Additional file 2: **Table S2.** The most enriched genes and proteins in the striated and catch adductor muscles of Yesso scallop *Patinopecten yessoensis*. List of the selected unigenes is divided into four categories, including muscle proteins, metabolism related enzymes, calcium signaling, membrane and extracellular proteins. The related information on quadrant (see Fig. 5), protein annotation, and false discovery rate (FDR) for these selected unigenes is summarized in this table. (DOC 60 kb)

## Abbreviations

BP: Biological process
CC: Cellular component
COG: Clusters of Orthologous Groups of proteins
DEGs: Differentially expressed genes
DEPs: Differentially expressed proteins
DTT: DL-Dithiothreitol
ECM: Extracellular matrix
E-LC: Myosin essential light chain
FDR: False discovery rate
GO: Gene Ontology
HPLC: High performance liquid chromatography
iTRAQ: Isobaric tags for relative and absolute quantitation
KEGG: Kyoto Encyclopedia of Genes and Genomes
KOG: euKaryotic Ortholog Groups
MF: Molecular function
MHC: Myosin heavy chain
Nr: NCBI non-redundant protein
PMSF: Phenylmethanesulfonyl fluoride
R-LC: Myosin regulatory light chain
SCP: Sarcoplasmic calcium-binding protein
SCX: Strong cation exchange
SERCA: Sarcoplasmic/endoplasmic reticulum calcium ATPase
SMTNL1: Moothelin-like 1 protein
SR: Sarcoplasmic reticulum
WEGO: Web Gene Ontology Annotation Plot
