# Supplementary material for: Differences between fast and slow muscles in scallops revealed through proteomics and transcriptomics
Source: BMC Genomics. 2018 May 22;19:377. doi: 10.1186/s12864-018-4770-2 (PMC5963113; doi:10.1186/s12864-018-4770-2)
Supplement: Supplementary file 2 — Table S2. The most enriched genes and proteins in the striated and catch adductor muscles of Yesso scallop Patinopecten yessoensis. List of the selected unigenes is divided into four categories, including muscle proteins, metabolism related enzymes, calcium signaling, membrane and extracellular proteins. The related information on quadrant (see Fig. 5), protein annotation, and false discovery rate (FDR) for these selected unigenes is summarized in this table. (DOC 60 kb) [file 12864_2018_4770_MOESM2_ESM.doc]

**Supplementary Table S1.** The most enriched genes and proteins in striated and smooth adductor muscle of scallops.

| **Category** | **GeneID** | **Quadrant** | **Protein annotation** | **Protein_FDR** | **mRNA_FDR** |
| --- | --- | --- | --- | --- | --- |
| Muscle proteins |  |  |  |  |  |
|  | Unigene0035479 | 1 | Filamin-A | 0.0643 | 0.0002 |
|  | Unigene0035486 | 1 | Filamin-C | 0.0663 | 0.0057 |
|  | Unigene0033782 | 3 | Alpha-actinin | 0.0086 | 0.0950 |
|  | Unigene0036106 | 3 | Enolase | 0.0455 | 0.0233 |
|  | Unigene0003983 | 3 | Muscle M-line assembly protein unc-89 | 0.0326 | 0.0000 |
|  | Unigene0007917 | 3 | Myosin heavy chain II | 0.0573 | 0.0008 |
|  | Unigene0003863 | 3 | PDZ and LIM domain protein 7 | 0.0515 | 0.0007 |
|  | Unigene0008319 | 3 | Titin | 0.0175 | 0.0219 |
|  | Unigene0035388 | 3 | Tropomodulin-1 | 0.0663 | 0.0005 |
|  | Unigene0017817 | 3 | Troponin C | 0.0663 | 0.0357 |
|  | Unigene0010779 | 3 | Troponin T | 0.0408 | 0.0107 |
|  | Unigene0026242 | 3 | Gelsolin-like protein 1 | 0.0375 | 0.0000 |
|  | Unigene0026243 | 3 | Gelsolin-like protein 2 | 0.0570 | 0.0002 |
|  | Unigene0040426 | 7 | LIM domain-containing protein | 0.0725 | 0.0109 |
|  | Unigene0035988 | 7 | Paramyosin | 0.0175 | 0.0474 |
|  | Unigene0033898 | 7 | PDZ and LIM domain protein 1 | 0.0477 | 0.0001 |
|  | Unigene0035464 | 7 | Actin-interacting protein 1 | 0.0507 | 0.0386 |
|  | Unigene0053787 | 7 | Calponin-3 | 0.0309 | 0.0647 |
|  | Unigene0011841 | 7 | Gelsolin-like protein 2 | 0.0564 | 0.0349 |
|  | Unigene0044504 | 7 | Myophilin | 0.0905 | 0.0213 |
|  | Unigene0035329 | 7 | Smoothelin-like protein 1 | 0.0437 | 0.0114 |
|  | Unigene0040865 | 7 | Twitchin | 0.0687 | 0.0000 |
| Metabolism related enzymes |  |  |  |  |  |
|  | Unigene0025019 | 1 | octopine dehydrogenase | 0.1523 | 0.3062 |
|  | Unigene0035573 | 3 | Calcium-transporting ATPase | 0.0542 | 0.0000 |
|  | Unigene0035820 | 3 | Glycogen debranching enzyme | 0.0661 | 0.0636 |
|  | Unigene0006324 | 3 | Glycogen phosphorylase | 0.0663 | 0.0004 |
|  | Unigene0003986 | 3 | Myosin light chain kinase | 0.0455 | 0.0000 |
|  | Unigene0035578 | 3 | Sarcoplasmic/endoplasmic reticulum calcium ATPase | 0.0375 | 0.0000 |
|  | Unigene0001573 | 3 | Phosphoglucomutase-1 | 0.1481 | 0.0170 |
|  | Unigene0003968 | 3 | Phosphoglucomutase-2 | 0.0694 | 0.0532 |
|  | Unigene0025975 | 3 | Pyruvate kinase | 0.0949 | 0.0006 |
|  | Unigene0035026 | 3 | Arginine kinase | 0.0468 | 0.0001 |
|  | Unigene0027790 | 6 | Glycogen synthase | 0.0826 | 1.0000 |
|  | Unigene0033106 | 7 | cAMP-dependent protein kinase regulatory subunit | 0.0365 | 0.0000 |
|  | Unigene0002490 | 7 | Glutathione S-transferase | 0.0861 | 0.0501 |
|  | Unigene0010886 | 7 | Glycogenin-1 | 0.0644 | 0.0553 |
| Calcium signaling |  |  |  |  |  |
|  | Unigene0041026 | 3 | 16 kDa calcium-binding protein | 0.0642 | 0.0081 |
|  | Unigene0024496 | 3 | Sarcoplasmic calcium-binding protein | 0.0574 | 0.0000 |
|  | Unigene0035556 | 7 | Sodium/calcium exchanger 3 | 0.0175 | 0.0000 |
| Membrane and extracellular proteins |  |  |  |  |  |
|  | Unigene0035530 | 1 | Laminin subunit gamma-1 | 0.0663 | 0.0563 |
|  | Unigene0008235 | 4 | perlecan | 0.0590 | 0.7169 |
|  | Unigene0025945 | 7 | Collagen alpha-5(VI) chain | 0.0175 | 0.0000 |
|  | Unigene0008262 | 7 | Laminin subunit alpha-1 | 0.0418 | 0.0012 |
|  | Unigene0008263 | 7 | Laminin subunit alpha-4 | 0.0570 | 0.0277 |
